# Inference of kinship using spatial distributions of SNPs for genome-wide association studies

**DOI:** 10.1186/s12864-016-2696-0

**Published:** 2016-05-20

**Authors:** Hyokyeong Lee, Liang Chen

**Affiliations:** Department of Biological Sciences, Molecular and Computational Biology, University of Southern California, Los Angeles, California 90089 USA

**Keywords:** GWAS, HapMap, Kinship, Population structure, SNP, 1000 Genomes

## Abstract

**Background:**

Genome-wide association studies (GWASs) are powerful in identifying genetic loci which cause complex traits of common diseases. However, it is well known that inappropriately accounting for pedigree or population structure leads to spurious associations. GWASs have often encountered increased type I error rates due to the correlated genotypes of cryptically related individuals or subgroups. Therefore, accurate pedigree information is crucial for successful GWASs.

**Results:**

We propose a distance-based method KIND to estimate kinship coefficients among individuals. Our method utilizes the spatial distribution of SNPs in the genome that represents how far each minor-allele variant is located from its neighboring minor-allele variants. The SNP distribution of each individual was presented in a feature vector in Euclidean space, and then the kinship coefficient was inferred from the two vectors of each individual pair. We demonstrate that the distance information can measure the similarity of genetic variants of individuals accurately and efficiently. We applied our method to a synthetic data set and two real data sets (i.e. the HapMap phase III and the 1000 genomes data). We investigated the estimation accuracy of kinship coefficients not only within homogeneous populations but also for a population with extreme stratification.

**Conclusions:**

Our method KIND usually produces more accurate and more robust kinship coefficient estimates than existing methods especially for populations with extreme stratification. It can serve as an important and very efficient tool for GWASs.

**Electronic supplementary material:**

The online version of this article (doi:10.1186/s12864-016-2696-0) contains supplementary material, which is available to authorized users.

## Background

Deciphering cryptic individual relatedness is crucial in genome-wide association studies (GWASs) because hidden population structure or cryptically related individuals result in false positives [1–6]. High-throughput technologies have enriched GWASs with millions of SNPs, and consequently pedigree errors can be corrected and cryptic familial relationships among samples can be detected. Those erroneous relationships could have been caused by the duplication of the genotypes at the time of data collection, artifacts, or undocumented relationships. Unexpected relationships were detected in the HapMap phase III data [7, 8]. Therefore, the relatedness inference should be performed in the data preprocessing step and be incorporated in GWAS algorithms.

Several methods have been proposed for relationship inference. These methods are mainly categorized into two types of approaches, likelihood methods [4, 9–11] and the kinship coefficient estimation methods [12–17]. The likelihood methods categorize pairwise relationships by choosing the most likely relationship among multiple relationships (i.e. monozygotic twin, parent-offspring, full sibling, *k*^*th*^-degree, or unrelated) while the kinship coefficient estimation methods quantify the pairwise relationship by calculating specific kinship coefficients. For example, the kinship coefficient is 0.25 for parent-offspring or full sibling relationship (note that the coefficient of relatedness is different and equals to 0.5 for the two cases). In the likelihood methods, the relationships of individuals are inferred using the likelihood of the Cotterman’s *k*-coefficients [9], the joint probability of the observed marker genotypes conditional on each potential relationship [10, 11], the conditional probabilities of identical-by-state (IBS) modes [4], or the conditional expected identical-by-descent (IBD) and adjusted identical-by-state (IBS) test [18]. For the kinship coefficient estimation methods, two sets of methods have been proposed. One set of methods attempts to reproduce the theoretical kinship coefficients [12–14]. The other set of methods produced their own kinship coefficients using IBD or IBS, and users could then specify coefficient cutoffs to declare relationships [15–17]. Our proposed method estimates the theoretical kinship coefficients as the former set of methods do, and it satisfies the urgent need of methods with high computational efficiency to handle the enormous number of SNPs from next-generation sequencing platforms.

Specifically, we propose an efficient method for kinship inference based on distance (KIND) to infer individual relationships. The proposed method is based on the SNP spatial distributions which show how far minor-allele variants are located one another. To the best of our knowledge, this is first time that the distance information of minor alleles was used in inferring pairwise relationships. We evaluated our model by comparing with KING (KING-robust version) [13] and REAP [14]. KING is a recently proposed kinship coefficient estimation method in the presence of population substructure. REAP is another recently proposed kinship estimation method for an admixed population. Note that many other kinship inference methods have the weakness of assuming homogeneous population structure. KING requires a full specification of pedigree structure for relationship estimation. The pedigree structure is then adjusted or corrected according to the inference. REAP does not need the family structure information and our KIND needs only a very small portion of the information. We studied a synthetic data set and two real data sets (i.e. the HapMap phase III and the 1000 genomes data).

## Methods

KIND is based on the spatial distribution of SNPs. For a specific individual, if there are two SNP sites both displaying minor alleles and there is no other SNP site displaying minor alleles between them, we call them neighboring minor-allele SNPs (or variants). The minor allele information was either obtained from the HapMap project directly or inferred by Plink [19] for each population. Given the genome of an individual, the physical distance between each minor-allele variant and its neighboring upstream minor-allele variant is calculated. The genomic-coordinate distance information together with the genotypes is used as a feature when estimating the kinship coefficient. The Minkowski distance (details in Additional file 1) is used to measure the dissimilarity of the feature vectors and then the dissimilarity score is converted to a kinship coefficient.

### Representation of alleles using the spatial distribution of variants

**Fig. 1:**
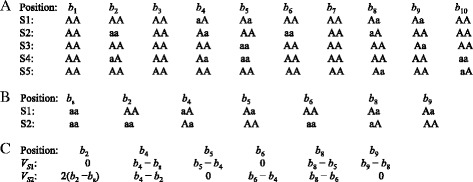
Flow from the SNPs to distance vectors. **a**: SNP distribution of a population of five individuals. **b**: SNP distribution after excluding positions containing only major alleles for the considered individual pair. Positions 1, 3, 7, and 10 are excluded and the initial position *b*_*s*_ is inserted manually. **c**: Distance vectors for individuals S1 and S2

Suppose that we have a set of genotype data of five individuals S1 – S5 (Fig. 1a) where “A” represents a major allele and “a” represents a minor allele for each SNP. We aim to infer the relationship between S1 and S2. The positions *b*_1_, *b*_2_, …, *b*_10_ are positions in base-pairs (bp) for SNP sites and 1 ≤ *b*_1_ < *b*_2_ < … < *b*_10_. The distance feature for a SNP position with minor alleles depends on the physical distance to its upstream neighboring minor-allele SNP. For example, an individual shows minor alleles at position *b*_*n*_ and its nearest upstream SNP position displaying minor alleles is *b*_*m*_. The distance feature for *b*_*n*_ is calculated as (*b*_*n*_ − *b*_*m*_) if both genotypes are Aa for the two positions, or one genotype is Aa and the other is aa. The distance feature is 2(*b*_*n*_ − *b*_*m*_) if both genotypes are aa since there are two neighboring minor alleles. Note that the distance feature is calculated regardless of phases. The distance feature for a SNP position with genotype AA is defined as zero. To avoid excessive zeros in features (the zeros will be cancelled out in distance calculation), positions with genotype AA for both individuals are excluded. Figure 1b shows the positions after removing *b*_1_, *b*_3_, *b*_7_ and *b*_10_ since no minor allele was observed at these positions for individuals S1 and S2. A position *b*_s_ with genotype “aa” was manually inserted as the initial position and it is the same across all the individuals in the population. We set the artificial initial position *b*_s_ as *b*_2_ − 10^−16^ where *b*_2_ is the first position with at least one minor allele among all the considered individuals in this specific example. This starting position *b*_s_ was assigned with the genotype “aa” so that different genotypes on the first minor-allele SNP can be distinguished. The feature vectors of individuals (*V*_S1_ and *V*_S2_ in Fig. 1c) are used to calculate the dissimilarity between S1 and S2. We chose the Minkowski distance to take advantage of the flexible order parameter of the distance function.

### Minkowski distance as a dissimilarity measure

Given two points *X* = (*x*_1_, *x*_2_, …, *x*_*n*_) and *Y* = (*y*_1_, *y*_2_, …, *y*_*n*_) (note that *V*_s1_ and *V*_s2_ above are examples of *X* and *Y*), the Minkowski distance between *X* and *Y* is defined as

1 $$ {\left({\displaystyle \sum_{i=1}^n\left|{x}_i-{y}_i\right|{}^p}\right)}^{1/p}. $$

The Minkowski distance is a generalization of the Euclidean (*p* = 2) and Manhattan (*p* = 1) distances [20, 21]. The order parameter *p* plays the key role in our proposed method in the sense that the optimal value of the parameter is determined by the underlying structure of the data and the estimated kinship coefficients are the presentation of the true relationships of individuals in the data.

### Similarity scores as kinship coefficients

In the relationship inference of two individuals, the dissimilarity score obtained from the distance function needs to be converted to a similarity score to represent a kinship coefficient. With the two distance vectors in Fig. 1c, the estimated kinship coefficient of individuals S_*i*_ and S_*j*_ is defined as

2 $$ \widehat{\varphi}=\left(1-\frac{d_{s_i,{s}_j}}{d_t}\right)/2, $$

where ${d}_{s_i,{s}_j}$ is the Minkowski distance between two individuals S_*i*_ and S_*j*_, *d*_*t*_ is a constant approximating the expected dissimilarity score of two unrelated individuals (More details in Additional file 1). If the two individuals S_*i*_ and S_*j*_have the exactly same allele distributions, $\widehat{\varphi}\kern0.5em  = 0.5$ because ${d}_{s_i,{s}_j}\kern0.5em  = 0$. For the self-kinship coefficient, $\widehat{\varphi}$ always equals 0.5. If the two individuals are unrelated, $\widehat{\varphi}\kern0.5em  = 0$ because ${d}_{s_i,{s}_j}\kern0.5em =\kern0.5em {d}_t$. Here *d*_*t*_ is a very rough estimate and it may be affected by minor allele frequencies, linkage disequilibrium, as well as deviation from Hardy-Weinberg Equilibrium. Note that the kindship estimation is not sensitive to *d*_*t*_ because we use training data to find the optimal *p* so that $\widehat{\varphi}\kern0.5em  = 0$ for unrelated individuals as described below.

### Optimal parameter estimation

The objective function is to minimize the sum of the squared errors between estimated kinship coefficients and theoretical kinship coefficients. Hence, the objective function is defined as

3 $$ \min\ {\displaystyle \sum_{\mathrm{Relationship}}{\left({\widehat{\varphi}}_{\mathrm{Relationship},p}-{\varphi}_{\mathrm{Relationship},p}\right)}^2}, $$

where available relationships in the data are used. In the results section, we show that a small amount of known data is sufficient for the optimal parameter estimation. Due to the nonlinearity of the objective function, nonlinear programming is applied to find the solution. Specifically, we applied a sequential quadratic programming (SQP) method which is a popular and successful nonlinear programming method [22]. A challenge associated with the optimization is that the search space of the unknown order parameter is infinite. Prior knowledge about the feasible search space significantly improves the convergence rate. After investigating the objective function values by using a number of parameter values with the HapMap phase III and the 1000 genomes data, we observed that a fairly good local minimum of the objective function was always between 0 and 1. Therefore, a feasible search space is provided by using the two boundaries of *p* as a constraint, i.e. 0 for the lower bound and 1 for upper bound. Finally, the SQP in line search is applied to

4 $$ \min\ {\displaystyle \sum_{\mathrm{Relationship}}{\left({\widehat{\varphi}}_{\mathrm{Relationship},p}-{\varphi}_{\mathrm{Relationship},p}\right)}^2}\kern0.5em \mathrm{subject}\ \mathrm{t}\mathrm{o}\ 0 < p\kern0.5em \le\ 1. $$

Note that the examples of the known relationships (*ϕ*_Relationship_) are *ϕ*_PO_ = 0.25 for a parent-offspring pair, *ϕ*_FS_ = 0.25 for a full sibling pair, *ϕ*_2nd_ = 0.125 for a 2^nd^–degree relative pair, *ϕ*_3rd_ = 0.0625 for a 3^rd^–degree relative pair, and *ϕ*_UN_ = 0 for an unrelated individual pair.

### Synthetic data generation

We simulated SNP data based on real data sets. Specifically we sampled 154 individuals from 7 non-inbred three-generation pedigrees as shown in Additional file 2: Figure S1. Unrelated CEU individuals of the 1000 genomes data were used for the top-most generation of each pedigree and the haplotypes were those from chromosome 19. For offspring at each generation, the chromosomes were recombined based on the recombination rates of chromosome 19 available in the 1000 genomes project data repository (http://www.1000genomes.org). Genotype data of the offspring were then simulated accordingly (a total of 250,182 SNPs).

## Results and discussion

### Consistency of estimated parameters with varying amount of known pedigree information

**Fig. 2:**
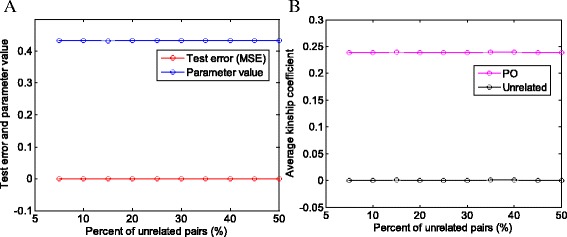
Results by varying number of unrelated individual pairs for training data. **a**: Test errors (mean squared errors) and estimated parameter values. The test errors were calculated on separated test data (the data excluding the training data). **b**: Average kinship coefficient estimates. Data: Chromosome 19 of the CEU samples in the HapMap phase III project

In real data analysis, the known pedigree information could be very limited. To test the accuracy of parameter estimation based on limited known pedigree structure, we used the CEU individual pairs of the HapMap phase III project. Different percentages of unrelated pairs (i.e. known to be unrelated individuals) were used for training. As shown in Fig. 2, the estimated parameter *p,* test errors (i.e. mean squared errors on test data), and estimated kinship coefficients are consistent across the different number of training pairs. We used only the chromosome 19 data (16,027 SNPs, the MAF distribution can be found in Additional file 3: Figure S2) but the similar results were observed when other autosomal chromosomes (chromosomes 1–18, 20–22), and other populations (YRI, CHB, and JPT) in the HapMap phase III and the 1000 genomes data were used (results not shown). The results confirm that a very small portion of known pedigree structure is enough for the estimation of the unknown model parameter *p.* In addition, a small training data set can significantly increase the computational efficiency.

### Relationship inference using synthetic data

Our proposed method was compared with the KING and the REAP software using the simulated synthetic data. There are two versions of KING. KING-homo estimates kinship coefficients of homogeneous populations and KING-robust is a more robust version and can handle population with extreme stratification (http://people.virginia.edu/~wc9c/KING/manual.html). Therefore KING-robust (a.k.a. KING) was used for the comparison. REAP infers kinship coefficients of populations with admixed ancestry. For the kinship coefficient estimation, REAP uses proportions and MAFs of ancestral populations for each individual that are inferred by the frappe software [23].

**Fig. 3:**
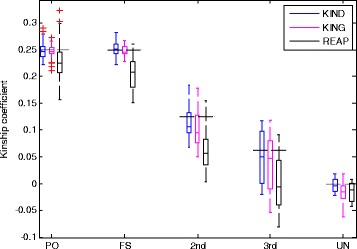
Kinship coefficient estimates for different types of family relationships based on synthetic data. The five horizontally dashed lines indicate the theoretical kinship coefficients of the five relationships (PO and FS: 0.25, 2^nd^: 0.125, 3^rd^: 0.0625, UN: 0). Data: 196 parent-offspring (PO) pairs, 49 full sibling (FS) pairs, 84 pairs at the 2nd degree (2nd), 56 pairs at the 3rd degree (3rd), and 18 unrelated (UN) pairs. For KIND, 10 % of UN pairs (i.e. 2 UN pairs) were used in estimating the unknown parameter

Figure 3 shows the boxplots of the estimated kinship coefficients of 196 parent-offspring (PO) pairs, 49 full sibling (FS) pairs, 84 pairs at the 2^nd^ degree (2^nd^), 56 pairs at the 3^rd^ degree (3^rd^), and 18 unrelated (UN) pairs. The detailed averages and standard deviations of the estimated kinship coefficients of the three methods were summarized in Additional file 4: Table S1. Our KIND estimates were usually closer to the theoretical kinship coefficients than the other two methods. The kinship coefficient estimates of REAP deviated a lot from the theoretical kinship coefficients. For the PO and FS pairs, the standard deviations of REAP were much larger than those of the other methods. For KIND, only two UN pairs (i.e. ~10 % of the UN pairs) were used for the parameter estimation. All other individuals except the two pairs were used for the test data. We also used two pairs from each of the five relationships for the parameter estimation. All other individuals except the 10 individual pairs were used for the test data. The results were similar as shown in Additional file 4: Table S1. It again suggests that using a small number of pairs even from a single relationship is enough to estimate the optimal value of the unknown parameter.

### Relationship inference using the HapMap phase III data

For the relationship inference on real data, we used the SNP data from the HapMap phase III. The selected populations were CEU (Utah residents with Northern and Western European ancestry, 165 individuals), YRI (Yoruba in Ibadan, Nigeria, 167 individuals), CHB (Han Chinese in Beijing, China, 84 individuals), and JPT (Japanese in Tokyo, Japan, 86 individuals). The autosomal chromosomes 1 through 22 were concatenated in the ascending order (chromosome1, chromosome2, …, chromosome22, 746,358-902,399 SNPs for these populations) for the kinship coefficient estimation. The available relationships of CEU and YRI are PO and UN, and only the UN relationship is available in CHB and JPT samples. The data consists of 96 PO and 6216 UN pairs for CEU, 104 PO and 6328 UN pairs for YRI, 3486 UN pairs for CHB, and 3655 UN pairs for JPT. The UN pairs of CEU and YRI populations were made from the PO data assuming that these parents were unrelated individuals. The number of ancestry populations for frappe and REAP was set to 3 and 5 % of the UN pairs were used in estimating the unknown parameter of our proposed method.

**Fig. 4:**
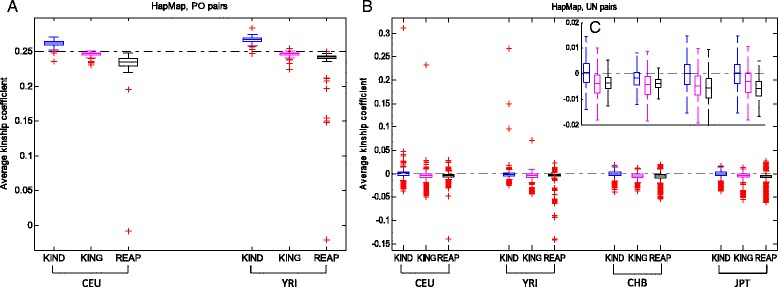
Boxplots of kinship coefficient estimates of the HapMap phase III data. **a**: PO pairs, **b**: UN pairs, **c**: UN pairs without plotting the outliers from B. The horizontally dashed lines indicate the theoretical kinship coefficients of the two relationships

Figure 4 shows the kinship coefficient estimates by the three methods for the PO (Fig. 4a) and UN pairs (Fig. 4b and c) in HapMap. Figure 4c is a subfigure to show the clearer quartiles of the estimates by not plotting those outliers in Fig. 4b. For UN pairs, KIND estimates were closer to the theoretical zero value for all considered populations compared with KING and REAP. For PO pairs, KIND performs slightly worse than KING. REAP misclassified some PO pairs as UN pairs because the estimated kinship was around zero instead of 0.25. Some extreme outliers existed in the KIND and KING estimates for CEU and YRI UN pairs. However, these outliers could be truly related pairs because we used parents from the parent-adult child data to make the UN pairs with the assumption that the parents of different family ids were unrelated. However, the parents from different families could be related. On the contrary, the CHB and JPT individuals are truly declared unrelated individuals. For both PO and UN pairs, the estimates of REAP were underestimated and usually with greater variance than those of the other two methods.

We observed that invalid estimations (NaN) were made by REAP for a large number of pairs, and some pairs were omitted in the results by KING. However, our KIND provides a valid estimate for every individual pair. Additional file 5: Table S2 shows the number of valid pairs per method, as well as the number of common pairs valid for all the three methods. REAP obtained invalid estimates for about half the PO pairs and KING missed estimates for about half the UN pairs in the YRI, CHB and JPT populations. We further investigated whether the different sample sizes affected the comparison results. When only the pairs with valid estimates for all three methods were considered, the average and standard deviation of the estimates were similar to those for all the pairs with valid estimates for each method (Additional file 6: Table S3 and Additional file 7: Table S4). KIND still performs the best for UN pairs and the performance is comparable for PO pairs.

### Relationship inference using the 1000 genomes data

**Fig. 5:**
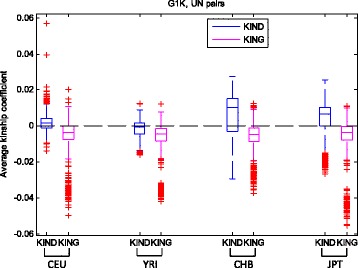
Boxplots of kinship coefficient estimates of the 1000 genomes data. The horizontally dashed line indicates the theoretical kinship coefficient (i.e. 0)

The same four populations were used for the 1000 genomes data and the samples were 85 CEU individuals, 88 YRI individuals, 97 CHB individuals, and 89 JPT individuals. The 1000 genomes project provides variant data only for unrelated individuals. A total number of 10,236,127-18,495,543 SNPs were used here for these populations. The total numbers of pairs are 3570 CEU pairs, 3828 YRI pairs, 4656 CHB pairs, and 3916 JPT pairs. About 5 % of the pairs were used in estimating the unknown parameter for each population in KIND. Because REAP failed in finishing the jobs in 300 hours (our Linux cluster walltime limit), we only summarized the results from KIND and KING. As shown in Fig. 5, KIND performs better than KING for CEU and YRI UN pairs, but not for CHB and JPT pairs. This may be due to the estimation of the parameter *p* being based on only unrelated individuals. KING has some extremely underestimated pairs for CHB and JPT. The specific number of pairs with valid estimates for each method or all methods, as well as the mean and standard deviation of the estimates are listed in Additional files 8, 9 and 10: Tables S5-S7.

### Relationship inference in the presence of population stratification

Estimating relatedness of individuals in the presence of population stratification is more complicated and challenging than the estimation for a homogeneous population. We made an artificial population by merging the four populations (CEU, YRI, CHB, and JPT) and then we assumed the population structure was unknown or hidden. The subgroups of similar SNP distributions were discovered by a clustering algorithm and then the within- and between-cluster kinship coefficients were estimated. Clusters of individuals were discovered by *k*-means clustering, the feature was the number of minor alleles at each base-pair position, and the dissimilarity measure was 1 - Pearson correlation. The 22 autosomal chromosomes were concatenated for the HapMap data and randomly selected three chromosomes (3, 18 and 20) were concatenated for the 1000 genomes data. In the experiment, the *k* was set to 1–5 and ten different initial cluster centers were tried per *k* because the clustering result depends on the initial cluster center. After replicating the clustering ten times per *k*, the set of clusters with minimum sum of point-centroid distances was selected and then the kinship coefficients for the within- and between-cluster were estimated. For the merged population from the HapMap data, YRI individuals were divided into two clusters, while CEU, CHB and JPT individuals formed one cluster respectively. For the merged population from the 1000 genomes data, two clusters from JPT and one cluster from each of the CEU, YRI, and CHB were discovered. Note that when estimating the between-cluster coefficients, individuals of the two clusters are combined for the estimation of optimal parameter *p*.

Estimating the optimal parameter value in the presence of population stratification was more difficult than that for homogeneous populations. When the population was homogeneous, only one local optimum was observed in the feasible search space and usually one initial guess was enough to find the local optimum. In the presence of population stratification, there may be more than one local optimum in the feasible search space. Multiple initial guesses were tried. When the solution was either the lower or upper bounds of the search space, the objective function value was noticeably large. In such cases, a different initial guess was tried and then the solution with the smaller objective function value was chosen. We estimated the within- and between-cluster kinship coefficients and then merged all of the estimated kinship coefficients for PO relationship or UN relationship.

**Fig. 6:**
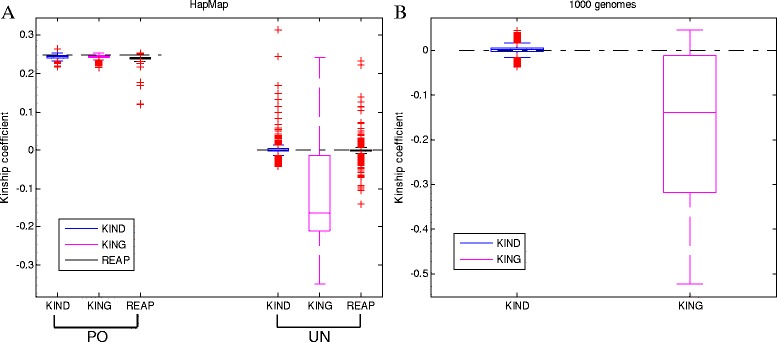
Boxplots of kinship coefficient estimates for the merged populations (CEU, YRI, CHB and JPT). **a**: HapMap individuals with PO or UN relationships, **b**: 1000 genomes individuals with UN relationship. The autosomal chromosomes were concatenated for the HapMap data but the randomly selected three chromosomes 3, 18, and 20 were concatenated for the 1000 genomes data to save computation time. The REAP result for the 1000 genomes data is not available because it did not finish within 300 hours of walltime. Individual pairs with valid estimates for all compared methods are used here. Specifically, the following steps were applied: Step 1. A set of clusters of individuals with similar minor allele distributions is discovered by the K-mean clustering. Step 2. For each cluster, the unknown order parameter is estimated (within-cluster estimation) and then the kinship coefficients for all individual pairs in the cluster are estimated. Step 3. For each pair of clusters, the parameter is estimated (between-cluster estimation) and then the kinship coefficients for all the individuals pairs, i.e. one individual from one cluster and the other individual from the other cluster, are estimated. Step 4. Combine the kinship coefficient estimates from Steps 2 and 3

Figure 6 shows the boxplots of the estimated kinship coefficients by KIND, KING and REAP for the mixed population based on the HapMap data (Fig. 6a) or the 1000 Genomes data (Fig. 6b). The averages and standard deviations of the kinship coefficients are in Additional file 11: Table S8. For this challenging situation, the performance of KIND is much better than KING and REAP by providing kinship coefficients closer to the theoretical values. KING heavily underestimated the kinship for UN pairs. The computation efficiency of REAP is unsatisfactory. Although only the three chromosomes were used from the 1000 genomes data, the result by REAP was not available because the frappe software failed to finish in the 300 hours of walltime.

### Comparison of computational complexity

Time complexity of the three methods is determined by the number of individuals and the number of SNPs. Let *n* and *m* be the number of individuals and the number of SNPs, respectively. The time complexity of KING is *O*(*n*^2^*m*). The time complexity of REAP consists of two parts. One part is the time complexity of frappe to infer proportions of each ancestral subpopulation for each individual and MAFs of each ancestral population. The time complexity of frappe is *O*(*mnkl*), where *k* is the number of ancestral subpopulations and *l* is the number of iterations of Expectation-Maximization (EM) algorithm. Theoretical time complexity of EM algorithm is infinite because the number of iterations *l* is not deterministic and it is highly dependent on the initial guess for the unknown parameters. To avoid the infinite iteration, frappe declares a convergence when the differences in the estimates of proportions and MAFs of ancestral subpopulations between two consecutive iterations fall below 10^−9^. The other part is the calculation of kinship coefficient of each individual pair by REAP using the proportions and MAFs of each ancestral subpopulation estimated by frappe, and the time complexity of this part is *O*(*n*^2^*mk*). Therefore, the total time complexity of REAP with frappe is O(*mnkl* + *n*^2^*mk*). The time complexity of our KIND is determined by the complexities of SQP and the calculation of kinship coefficient. The time complexity of SQP is determined by the number of SQP iterations and the quadratic programming (QP) subproblem iterations. SQP solves QP subproblems at every iteration. The QP is also an iterative method and the number of QP iterations is determined by the number of inequality constraints which is 1 in our proposed method because the number of unknown parameter is only 1, i.e. 0 < *p* ≤ 1. Similar to EM algorithm, SQP is also an iterative method and a convergence is declared when the difference of two objective function values between two consecutive iterations falls below 10^−6^ or the SQP iterations reach 400. Therefore, the time complexity of SQP is *O*(*l*), where *l* is the number of SQP iteration. The time complexity of the kinship coefficient calculation is *O*(*n*^2^*m*). Therefore, the total time complexity of our KIND is *O*(*l*(*n’*)^2^*m* + *n*^2^*m*), where *n’* is the number of individuals in the training data and *n’* < < *n*. It is slightly larger than that of KING, but much more efficient than REAP.

We measured the elapsed time on Intel Xeon E5-2665 2.4 GHz processor with 40GB of memory. The elapsed time of KING was the least among the three methods and that of REAP took the longest because of frappe. KIND has a slightly longer computation time than KING. The elapsed time of frappe significantly increased as the number of SNPs increased. The frappe software finished in 61 hours for the HapMap data but did not finish in 300 hours which were the maximum allowed running hours in the experiments. There may be strategies which would allow high density SNP data to be used with REAP.

## Discussions and conclusions

We developed a distance-based method KIND to infer pairwise relationships of individuals for GWASs. Our method uses spatial distribution information of SNPs. First, we found that the minor-allele distance information reveals the relationships of individuals. Existing methods used numbers of shared alleles at loci or probability of marker data conditional on each relationship. We showed that the distance information is able to identify the pairwise relationships. As we know, recombination hotspots are distributed along the human genome unevenly [24]. Since recombination is the main cause of genetic diversity, the hotspot distribution leads to unequal physical distances between neighboring SNPs. Based on this fact, we suspect that the distance between genetic variants has left footprints for kinship inference. Here, we calculated the minor-allele distance between SNPs. In the future studies, we can further test the average distance to neighboring SNPs or incorporate the MAF-based weighting strategy in the distance calculation. Second, a fairly good value for the unknown model parameter was found by using a small amount of training data. This enables the efficiency of our method.

When the order parameter of the Minkowski distance is greater than 0 and smaller than 1, the triangle inequality does not hold and it is called a semi-metric. The loss of triangle inequality did not affect negatively on the kinship estimation. Using both of the HapMap and 1000 genomes data, we plotted the values of the objective function (Eq 3) using the *p* values between −1 and 5. The global minimum was always observed and the optimal value of *p* was found between 0 and 1.

The kinship coefficient estimation from high throughput genotype data is still challenging. Different methods obtained different estimates. As shown in Additional file 12: Table S9, our KIND estimates have a correlation of 0.53 (UN) or 0.74 (PO) with KING estimates, but only a correlation of 0.11 (UN) or 0.36 (PO) with REAP estimates. KING and REAP estimates are very different with a correlation of only 0.09 (UN) and 0.16 (PO). When combining the UN and the PO relationships for the correlation calculation, REAP still shows the least correlation with other two methods (Additional file 12: Table S9).

The boxplots of the estimated kinship coefficients of UN pairs show some negative coefficients. In the case of KIND, negative values are generated when *d*_*si,sj*_ is greater than *d*_*t*_. Negative values are observed in the coefficients estimated by KING and REAP as well. In the case of KING, when two individuals in a homogeneous population are unrelated, the coefficient is negative. In the event that a pair of individuals is drawn from two distinct populations, the coefficient by KING is extremely smaller than that of a pair of individuals drawn from a homogeneous population [13]. In the case of REAP, the negative values are observed in the pairs of not only unrelated but also 3^rd^ degree relatives.

Based on the analysis on synthetic and real data, we found that our KIND performs better especially when populations are mixed. Although our KIND is not especially designed for heterogeneous population, we found that KIND is robust to the heterogeneity due to its flexibility and simplicity. Our KIND simply focuses on “variants” of each individual. If we used “major” alleles instead of “minor” alleles in our distance feature calculation, the results are similar (Additional file 13: Table S10). If we consider “pruned” set of SNPs (i.e. SNPs with MAF > 0.4) in KIND, the kinship estimation is similar to that with all SNPs (Additional file 14: Table S11). This further indicates that our kinship estimation is not sensitive to the minor allele frequency cutoff of SNPs. For the population stratification, we simply applied the K-mean clustering to subdivide the data so that KIND can be applied. More sophisticated models can be applied in future studies.

## Additional files

Additional file 1: Supplementary text. (DOCX 43 kb) Additional file 2: Figure S1. Pedigree configuration of the synthetic data. (DOC 55 kb) Additional file 3: Figure S2. The distribution of minor allele frequencies of SNPs on chr 19 of the HapMap CEU individuals. (DOC 26 kb) Additional file 4: Table S1. Average (standard deviation) of kinship coefficients of synthetic data by each method and the estimated value of the unknown parameter p for KIND, PO: parent-offspring, FS: full-sibling, 2nd: 2nd degree, 3rd: 3rd degree, UN: unrelated. (DOC 30 kb) Additional file 5: Table S2. Number of all valid pairs per population and method, and common pairs valid for the three methods. Data: HapMap phase III. (DOC 29 kb) Additional file 6: Table S3. Average (standard deviation) of kinship coefficient estimates of the three methods for all valid pairs and the estimated values of the unknown parameter p for KIND. Data: HapMap phase III. (DOC 32 kb) Additional file 7: Table S4. Average (standard deviation) of kinship coefficient estimates of the three methods for common valid pairs and the estimated values of the unknown parameter p for KIND. Data: HapMap phase III. (DOC 32 kb) Additional file 8: Table S5. Number of all valid pairs per population and method, and common valid pairs for KIND and KING. Data: 1000 genomes. Note: Kinship coefficient estimates by REAP are not available because frappe did not finish within the 300 hour walltime. (DOC 28 kb) Additional file 9: Table S6. Average (standard deviation) of kinship coefficient estimates of KIND and KING for all valid pairs, and the estimated values of the unknown parameter p for KIND. Data: 1000 genomes. Note: Kinship coefficient estimates by REAP are not available because frappe did not finish within the 300 hour walltime. (DOC 29 kb) Additional file 10: Table S7. Average (standard deviation) of kinship coefficient estimates of KIND and KING for common pairs and the estimated values of the unknown parameter p for KIND. Data: 1000 genomes. Note: Kinship coefficient estimates by REAP are not available because frappe did not finish within the 300 hour walltime. (DOC 29 kb) Additional file 11: Table S8. Average (standard deviation) of kinship coefficient estimates for common pairs among KIND, KING, and REAP. Data: merged CEU, YRI, CHB, and JPT from HapMap phase III and 1000 genomes phase III, respectively. Note: Kinship coefficient estimates by REAP are not available for 1000 genomes because frappe did not finish within the 300 hour walltime. (DOC 29 kb) Additional file 12: Table S9. Correlation coefficients of kinship coefficient estimates by different methods. Chromosomes 1–22 of HapMap CEU data were used. (DOC 28 kb) Additional file 13: Table S10. Kinship estimation by KIND using “major” or “minor” alleles in features. HapMap CEU population data were used. The average and standard deviation of the estimates are shown. (DOC 28 kb) Additional file 14: Table S11. Kinship estimation using SNPs with MAF >0.4. Data are from the CEU population of 1000 genomes data. The averages (and standard deviation) of kinship coefficient estimates are shown. (DOC 28 kb)

## Abbreviations

GWASs: genome-wide association studies
KIND: kinship inference based on distance
SNP: Single Nucleotide Polymorphism
CEU: Utah residents with ancestry from northern and western Europe
YRI: Yoruba in Ibadan, Nigeria
CHB: Han Chinese in Beijing, China
JPT: Japanese in Tokyo, Japan
PO: parent-offspring
FS: full sibling
UN: unrelated
