# Supplementary material for: Inference of kinship using spatial distributions of SNPs for genome-wide association studies
Source: BMC Genomics. 2016 May 20;17:372. doi: 10.1186/s12864-016-2696-0 (PMC4873983; doi:10.1186/s12864-016-2696-0)
Supplement: Additional file 1: — Supplementary text. (DOCX 43 kb) [file 12864_2016_2696_MOESM1_ESM.docx]

**Additional file 1:** Supplementary text

**Minkowski inequality**
A function is a metric because it satisfies the four conditions as following. Let *d*(∙) be the Minkowski distance between two points.

1. Non-negativity: *d*(*x*, *y*) ≥ 0

2. Identity of indiscernibles: *d*(*x*, *y*) = 0 iff *x* = *y*

3. Symmetry: *d*(*x*, *y*) = *d*(*y*, *x*)

4. Subadditivity/Triangle inequality: *d*(*x*, *y*) ≤ *d*(*x*, *z*) + *d*(*y*, *z*)

When the subadditivity does not hold, *d* is semi-metric. The order parameter *p* the Minkowski distance determines whether the Minkowski inequality holds or not (Thompson 1996; Voitsekhovskii 2001). If *x*, *y* are in **R***^d^* and *p* > 1, then ||*x* + *y*||*_p_* ≤ ||*x*||*_p_* + ||*y*||*_p_*. For *p* = 2, Minkowski inequality is called the triangle inequality. For 0 < *p* < 1, the inequality is reversed and the Minkowski distance is a semi-metric.

**Expected dissimilarity score for two unrelated individuals**

In calculating the kinship coefficients, *d_t_* is the dissimilarity score that represents the unrelated relationship in the population. To simplify the calculation and apply a single *d_t_* value for all individual pairs, we consider all SNP positions. Let *d* be the physical distance (in bp) between two neighboring positions *r* and *s* (*r* < *s*). The distance feature for *s* is 0, *d*, or 2*d* depending on the existence of minor alleles at the two positions. Thus, the probability that the difference feature is 0, *d*, or 2*d* are defined as

$\Delta_{0}=\Pr(\text{distance} \text{feature}=0)=p_{r}^{2}+(1-p_{r}^{2})p_{s}^{2}$,

$\Delta_{d}=\Pr(\text{distance} \text{feature}=d)=2p_{r}(1-p_{r})(1-p_{s}^{2})+(1-p_{r})^{2}2p_{s}(1-p_{s})$,

$\Delta_{2d}=\Pr(\text{distance} \text{feature}=2d)=(1-p_{r})^{2}(1-p_{s})^{2}$,

where *p_r_* and *p_s_* are the major allele frequencies at *r* and *s*, respectively. Considering the difference of the distance features between two unrelated individuals at *s* (denoted as *dd*_s_), their probabilities are

$\Pr(dd_{s}=0)=(\Delta_{0}^{2}+\Delta_{d}^{2}+\Delta_{2d}^{2}-p_{r}^{4}-p_{s}^{4}+p_{r}^{4}p_{s}^{4})/\alpha_{\text{within}}$,

$\Pr(dd_{s}=d)=(2\Delta_{0}\Delta_{d}+2\Delta_{d}\Delta_{2d})/\alpha_{\text{within}}$,

$\Pr(dd_{s}=2d)=2\Delta_{0}\Delta_{2d}/\alpha_{\text{within}}$,

where $\alpha_{\text{within}}=1-p_{r}^{4}-p_{s}^{4}+p_{r}^{4}p_{s}^{4}$ and is a normalization factor so that the sum of the probability is one.

Note that:

1. *dd*_s_ =0 when the distance features for individual 1 and individual 2 are (0,0), (d,d), or (2d,2d).
2. *dd*_s_ =d when the distance features for individual 1 and individual 2 are (0,d),(d,0),(d,2d), or (2d,d)
3. *dd*_s_ =2d when the distance features for individual 1 and individual 2 are (0,2d) or (2d,0).

The above probabilities related to *dd*_s_ are then calculated based on the enumerated scenarios.

Thus, $E(dd_{s})=0*\Pr(dd_{s}=0)+d*\Pr(dd_{s}=d)+2d*\Pr(dd_{s}=2d)$.

Therefore, in the example of Figure 1,

$d_{s_{1},s_{2}}=(|0-2(b_{2}-b_{s})|^{p}+|(b_{4}-b_{s})-(b_{4}-b_{2})|^{p}+...+|(b_{9}-b_{8})-0|^{p})^{1/p}$and

$$d_{t}=(E(dd_{1})^{p}+E(dd_{4})^{p}+\cdots+(E(dd_{10})^{p})^{1/p}$$

Although *d_t_* is a very rough estimate and it may be affected by minor allele frequencies, linkage disequilibrium, as well as deviation from Hardy-Weinberg Equilibrium, the kinship estimation is not sensitive to the *d_t_* estimate because we use training data to find the optimal *p* so that $\hat{\varphi}$ = 0 for unrelated individuals.
