# Supplementary figures and images for: Inference of kinship using spatial distributions of SNPs for genome-wide association studies
Source: BMC Genomics. 2016 May 20;17:372. doi: 10.1186/s12864-016-2696-0 (PMC4873983; doi:10.1186/s12864-016-2696-0)

**Additional file 2**

Figure S1. Pedigree configuration of the synthetic data.


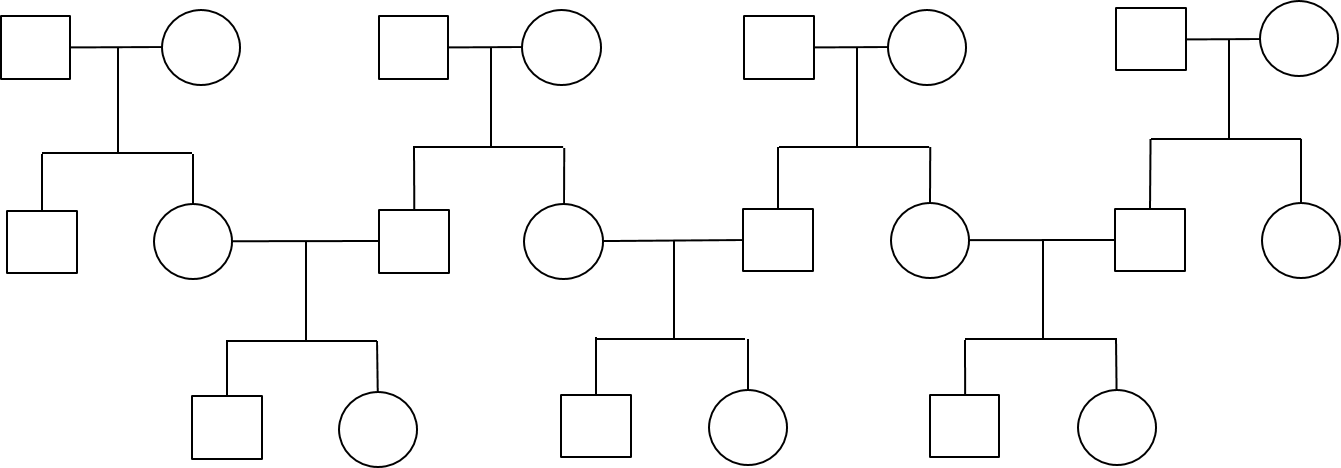

Supplement: Additional file 2: Figure S1. — Pedigree configuration of the synthetic data. (DOC 55 kb) [file 12864_2016_2696_MOESM2_ESM.doc]
