# Supplementary material for: Inference of kinship using spatial distributions of SNPs for genome-wide association studies
Source: BMC Genomics. 2016 May 20;17:372. doi: 10.1186/s12864-016-2696-0 (PMC4873983; doi:10.1186/s12864-016-2696-0)
Supplement: Additional file 3: Figure S2. — The distribution of minor allele frequencies of SNPs on chr 19 of the HapMap CEU individuals. (DOC 26 kb) [file 12864_2016_2696_MOESM3_ESM.doc]

**Additional file 3**

Figure S2. The distribution of minor allele frequencies of SNPs on chr 19 of the HapMap CEU individuals.
