# Supplementary material for: Inference of kinship using spatial distributions of SNPs for genome-wide association studies
Source: BMC Genomics. 2016 May 20;17:372. doi: 10.1186/s12864-016-2696-0 (PMC4873983; doi:10.1186/s12864-016-2696-0)
Supplement: Additional file 4: Table S1. — Average (standard deviation) of kinship coefficients of synthetic data by each method and the estimated value of the unknown parameter p for KIND, PO: parent-offspring, FS: full-sibling, 2nd: 2nd degree, 3rd: 3rd degree, UN: unrelated. (DOC 30 kb) [file 12864_2016_2696_MOESM4_ESM.doc]

**Additional file 4**

Table S1. Average (standard deviation) of kinship coefficients of synthetic data by each method and the estimated value of the unknown parameter *p* for KIND, PO: parent-offspring, FS: full-sibling, 2^nd^: 2^nd^ degree, 3^rd^: 3^rd^ degree, UN: unrelated.

| Relationship (theoretical kinship coefficient) | KIND | | KING | REAP |
| --- | --- | --- | --- | --- |
|  | 2 pairs from each relationship (*p* = 0.5052) | 2 UN pairs (*p* = 0.5066) |  |  |
| PO (0.25) | 0.2497 (0.0130) | 0.2472 (0.0130) | 0.2470 (0.0093) | 0.2256 (0.0310) |
| FS (0.25) | 0.2534 (0.0136) | 0.2510 (0.0137) | 0.2481 (0.0090) | 0.2055 (0.0279) |
| 2^nd^ (0.125) | 0.1198 (0.0295) | 0.1166 (0.0296) | 0.1028 (0.0324) | 0.0636 (0.0393) |
| 3^rd^ (0.0625) | 0.0530 (0.0485) | 0.0496 (0.0487) | 0.0365 (0.0518) | -0.0007 (0.0484) |
| UN (0) | 0.0010 (0.0119) | -0.0027 (0.0120) | -0.0160 (0.0204) | -0.0144 (0.0171) |
