# Supplementary material for: Inference of kinship using spatial distributions of SNPs for genome-wide association studies
Source: BMC Genomics. 2016 May 20;17:372. doi: 10.1186/s12864-016-2696-0 (PMC4873983; doi:10.1186/s12864-016-2696-0)
Supplement: Additional file 5: Table S2. — Number of all valid pairs per population and method, and common pairs valid for the three methods. Data: HapMap phase III. (DOC 29 kb) [file 12864_2016_2696_MOESM5_ESM.doc]

**Additional file 5**

Table S2. Number of all valid pairs per population and method, and common pairs valid for the three methods. Data: HapMap phase III.

|  | CEU | | | | YRI | | | |
| --- | --- | --- | --- | --- | --- | --- | --- | --- |
|  | All | | | Common | All | | | Common |
| Relationship | KIND | KING | REAP |  | KIND | KING | REAP |  |
| PO | 96 | 89 | 47 | 44 | 104 | 104 | 54 | 54 |
| UN | 6216 | 6190 | 6216 | 6190 | 6328 | 3494 | 6328 | 3494 |
|  | CHB | | | | JPT | | | |
|  | All | | | Common | All | | | Common |
| Relationship | KIND | KING | REAP |  | KIND | KING | REAP |  |
| UN | 3486 | 1618 | 3486 | 1618 | 3655 | 1595 | 3655 | 1595 |
