# Supplementary material for: Inference of kinship using spatial distributions of SNPs for genome-wide association studies
Source: BMC Genomics. 2016 May 20;17:372. doi: 10.1186/s12864-016-2696-0 (PMC4873983; doi:10.1186/s12864-016-2696-0)
Supplement: Additional file 6: Table S3. — Average (standard deviation) of kinship coefficient estimates of the three methods for all valid pairs and the estimated values of the unknown parameter p for KIND. Data: HapMap phase III. (DOC 32 kb) [file 12864_2016_2696_MOESM6_ESM.doc]

**Additional file 6**

Table S3. Average (standard deviation) of kinship coefficient estimates of the three methods for all valid pairs and the estimated values of the unknown parameter *p* for KIND. Data: HapMap phase III

|  | CEU | | | YRI | | |
| --- | --- | --- | --- | --- | --- | --- |
| Relationship | KIND (*p* = 0.4570) | KING | REAP | KIND (*p* = 0.4349) | KING | REAP |
| PO | 0.2621 (0.0051) | 0.2462 (0.0039) | 0.2290 (0.0364) | 0.2672 (0.0047) | 0.2467 (0.0037) | 0.2302 (0.0416) |
| UN | -0.0002 (0.0076) | -0.0050 (0.0086) | -0.0038 (0.0058) | -0.0021 (0.0060) | -0.0054 (0.0068) | -0.0039 (0.0057) |
|  | CHB | | | JPT | | |
| Relationship | KIND (*p* = 0.4741) | KING | REAP | KIND (*p* = 0.4691) | KING | REAP |
| UN | -0.0010 (0.0069) | -0.0050 (0.0061) | -0.0060 (0.0088) | -0.0007 (0.0069) | -0.0046 (0.0086) | -0.0059 (0.0086) |
