# Supplementary material for: Inference of kinship using spatial distributions of SNPs for genome-wide association studies
Source: BMC Genomics. 2016 May 20;17:372. doi: 10.1186/s12864-016-2696-0 (PMC4873983; doi:10.1186/s12864-016-2696-0)
Supplement: Additional file 7: Table S4. — Average (standard deviation) of kinship coefficient estimates of the three methods for common valid pairs and the estimated values of the unknown parameter p for KIND. Data: HapMap phase III. (DOC 32 kb) [file 12864_2016_2696_MOESM7_ESM.doc]

**Additional file 7**

Table S4. Average (standard deviation) of kinship coefficient estimates of the three methods for common valid pairs and the estimated values of the unknown parameter *p* for KIND. Data: HapMap phase III

|  | CEU | | | YRI | | |
| --- | --- | --- | --- | --- | --- | --- |
| Relationship | KIND (*p* = 0.4570) | KING | REAP | KIND (*p* = 0.4352) | KING | REAP |
| PO | 0.2626 (0.0036) | 0.2463 (0.0038) | 0.2286 (0.0376) | 0.2667 (0.0052) | 0.2467 (0.0034) | 0.2302 (0.0416) |
| UN | -0.0002 (0.0076) | -0.0050 (0.0086) | -0.0038 (0.0056) | -0.0030 (0.0051) | -0.0054 (0.0068) | -0.0037 (0.0052) |
|  | CHB | | | JPT | | |
| Relationship | KIND (*p* = 0.4739) | KING | REAP | KIND (*p* = 0.4693) | KING | REAP |
| UN | -0.0007 (0.0074) | -0.0050 (0.0061) | -0.0061 (0.0096) | -0.0010 (0.0068) | -0.0046 (0.0086) | -0.0056 (0.0087) |
