# Supplementary material for: Inference of kinship using spatial distributions of SNPs for genome-wide association studies
Source: BMC Genomics. 2016 May 20;17:372. doi: 10.1186/s12864-016-2696-0 (PMC4873983; doi:10.1186/s12864-016-2696-0)
Supplement: Additional file 8: Table S5. — Number of all valid pairs per population and method, and common valid pairs for KIND and KING. Data: 1000 genomes. Note: Kinship coefficient estimates by REAP are not available because frappe did not finish within the 300 hour walltime. (DOC 28 kb) [file 12864_2016_2696_MOESM8_ESM.doc]

**Additional file 8**

Table S5. Number of all valid pairs per population and method, and common valid pairs for KIND and KING. Data: 1000 genomes. Note: Kinship coefficient estimates by REAP are not available because frappe did not finish within the 300 hour walltime.

| CEU | | | YRI | | | CHB | | | JPT | | |
| --- | --- | --- | --- | --- | --- | --- | --- | --- | --- | --- | --- |
| All | | Common | All | | Common | All | | Common | All | | Common |
| KIND | KING |  | KIND | KING |  | KIND | KING |  | KIND | KING |  |
| 3570 | 2691 | 2691 | 3828 | 3228 | 3228 | 4656 | 4656 | 4656 | 3916 | 3916 | 3916 |
