# Supplementary material for: Inference of kinship using spatial distributions of SNPs for genome-wide association studies
Source: BMC Genomics. 2016 May 20;17:372. doi: 10.1186/s12864-016-2696-0 (PMC4873983; doi:10.1186/s12864-016-2696-0)
Supplement: Additional file 9: Table S6. — Average (standard deviation) of kinship coefficient estimates of KIND and KING for all valid pairs, and the estimated values of the unknown parameter p for KIND. Data: 1000 genomes. Note: Kinship coefficient estimates by REAP are not available because frappe did not finish within the 300 hour walltime. (DOC 29 kb) [file 12864_2016_2696_MOESM9_ESM.doc]

**Additional file 9**

Table S6. Average (standard deviation) of kinship coefficient estimates of KIND and KING for all valid pairs, and the estimated values of the unknown parameter *p* for KIND. Data: 1000 genomes. Note: Kinship coefficient estimates by REAP are not available because frappe did not finish within the 300 hour walltime.

| CEU | | YRI | | CHB | | JPT | |
| --- | --- | --- | --- | --- | --- | --- | --- |
| KIND (*p* = 0.5264) | KING | KIND (*p* = 0.5157) | KING | KIND (*p* = 0.5331) | KING | KIND (*p* = 0.5276) | KING |
| 0.0017 (0.0043) | -0.0072 (0.0073) | -0.0018 (0.0047) | -0.0035 (0.0040) | 0.0068 (0.0110) | -0.0086 (0.0078) | 0.0039 (0.0095) | -0.0089 (0.0148) |
