# Supplementary material for: Inference of kinship using spatial distributions of SNPs for genome-wide association studies
Source: BMC Genomics. 2016 May 20;17:372. doi: 10.1186/s12864-016-2696-0 (PMC4873983; doi:10.1186/s12864-016-2696-0)
Supplement: Additional file 11: Table S8. — Average (standard deviation) of kinship coefficient estimates for common pairs among KIND, KING, and REAP. Data: merged CEU, YRI, CHB, and JPT from HapMap phase III and 1000 genomes phase III, respectively. Note: Kinship coefficient estimates by REAP are not available for 1000 genomes because frappe did not finish within the 300 hour walltime. (DOC 29 kb) [file 12864_2016_2696_MOESM11_ESM.doc]

**Additional file 11**

Table S8. Average (standard deviation) of kinship coefficient estimates for common pairs among KIND, KING, and REAP. Data: merged CEU, YRI, CHB, and JPT from HapMap phase III and 1000 genomes phase III, respectively. Note: Kinship coefficient estimates by REAP are not available for 1000 genomes because frappe did not finish within the 300 hour walltime.

| Data | Relationship | KIND | KING | REAP |
| --- | --- | --- | --- | --- |
| HapMap | PO | 0.2437 (0.0059) | 0.2440 (0.0058) | 0.2387 (0.0144) |
|  | UN | -0.0002 (0.0058) | -0.1449 (0.1042) | -0.0014 (0.0036) |
| 1000 genomes | UN | 0.0009 (0.0068) | -0.1622 (0.1423) | ― |
