# Supplementary material for: Inference of kinship using spatial distributions of SNPs for genome-wide association studies
Source: BMC Genomics. 2016 May 20;17:372. doi: 10.1186/s12864-016-2696-0 (PMC4873983; doi:10.1186/s12864-016-2696-0)
Supplement: Additional file 12: Table S9. — Correlation coefficients of kinship coefficient estimates by different methods. Chromosomes 1–22 of HapMap CEU data were used. (DOC 28 kb) [file 12864_2016_2696_MOESM12_ESM.doc]

**Additional file 12**

Table S9. Correlation coefficients of kinship coefficient estimates by different methods. Chromosomes 1 - 22 of HapMap CEU data were used.

| Relationship | KIND vs. KING | KIND vs. REAP | KING vs. REAP |
| --- | --- | --- | --- |
| PO | 0.7371 | 0.3601 | 0.1640 |
| UN | 0.5329 | 0.1073 | 0.0902 |
| PO and UN combined | 0.9685 | 0.9102 | 0.8894 |
