# Supplementary material for: Inference of kinship using spatial distributions of SNPs for genome-wide association studies
Source: BMC Genomics. 2016 May 20;17:372. doi: 10.1186/s12864-016-2696-0 (PMC4873983; doi:10.1186/s12864-016-2696-0)
Supplement: Additional file 13: Table S10. — Kinship estimation by KIND using “major” or “minor” alleles in features. HapMap CEU population data were used. The average and standard deviation of the estimates are shown. (DOC 28 kb) [file 12864_2016_2696_MOESM13_ESM.doc]

**Additional file 13**

Table S10. Kinship estimation by KIND using “major” or “minor” alleles in features. HapMap CEU population data were used. The average and standard deviation of the estimates are shown.

|  | Relationship | KIND |
| --- | --- | --- |
| Minor alleles | PO | 0.2621 (0.0051) |
|  | UN | −0.0002 (0.0076) |
| Major alleles | PO | 0.2477 (0.0050) |
|  | UN | 0.0046 (0.0057) |
