# Supplementary material for: Inference of kinship using spatial distributions of SNPs for genome-wide association studies
Source: BMC Genomics. 2016 May 20;17:372. doi: 10.1186/s12864-016-2696-0 (PMC4873983; doi:10.1186/s12864-016-2696-0)
Supplement: Additional file 14: Table S11. — Kinship estimation using SNPs with MAF >0.4. Data are from the CEU population of 1000 genomes data. The averages (and standard deviation) of kinship coefficient estimates are shown. (DOC 28 kb) [file 12864_2016_2696_MOESM14_ESM.doc]

**Additional file 14**

Table S11. Kinship estimation using SNPs with MAF >0.4. Data are from the CEU population of 1000 genomes data. The averages (and standard deviation) of kinship coefficient estimates are shown.

|  | KIND | KING | REAP |
| --- | --- | --- | --- |
| UN (MAF > 0) | 0.0017 (0.0043) | −0.0072 (0.0073) | * |
| UN (MAF > 0.4) | 0.0007 (0.0090) | −0.0071 (0.0109) | −0.0058 (0.0094) |

*REAP did not finish within the walltime (300 hours) when all the SNPs were used.
